# DNA methylation as a triage marker for colposcopy referral in HPV-based cervical cancer screening: a systematic review and meta-analysis

**DOI:** 10.1186/s13148-023-01537-2

**Published:** 2023-08-02

**Authors:** Sofia Salta, João Lobo, Bruno Magalhães, Rui Henrique, Carmen Jerónimo

**Affiliations:** 1 Cancer Biology & Epigenetics Group, Research Center of IPO Porto (CI-IPOP) / RISE@CI-IPOP (Health Research Network), Portuguese Oncology Institute of Porto (IPO Porto) / Porto Comprehensive Cancer Center Raquel Seruca (Porto.CCC), Rua Dr. António Bernardino de Almeida, 4200-072 Porto, Portugal; 2 Doctoral Program in Molecular Pathology and Genetics, School of Medicine & Biomedical Sciences (ICBAS-UP), Rua de Jorge Viterbo Ferreira, 228, 4050-313 Porto, Portugal; 3 ESS, Polytechnic of Porto, Rua Dr. António Bernardino de Almeida, 400, 4200-072 Porto, Portugal; 4 Department of Pathology, Portuguese Oncology Institute of Porto (IPO Porto), Rua Dr. António Bernardino de Almeida, 4200-072 Porto, Portugal; 5 Department of Pathology and Molecular Immunology, School of Medicine and Biomedical Sciences (ICBAS-UP), Rua Jorge Viterbo Ferreira 228, 4050-513 Porto, Portugal; 6 Oncology Nursing Research Unit, Research Center of IPO Porto (CI-IPOP) /CI-IPOP@RISE (Health Research Network), Portuguese Oncology Institute of Porto (IPO Porto) / Porto Comprehensive Cancer Center Raquel Seruca (Porto.CCC), Rua Dr. António Bernardino de Almeida, 4200-072 Porto, Portugal; 7 School of Health, University of Trás-os-Montes and Alto Douro, Vila Real, Portugal; 8 Clinical Academic Centre of Trás-os-Montes and Alto Douro (CACTMAD), Vila Real, Portugal

**Keywords:** Cervical cancer screening, Triage, DNA methylation, Biomarkers, Colposcopy referral

## Abstract

**Background:**

Screening plays a key role in secondary prevention of cervical cancer. High-risk human papillomavirus (hrHPV) testing, a highly sensitive test but with limited specificity, has become the gold standard frontline for screening programs. Thus, the importance of effective triage strategies, including DNA methylation markers, has been emphasized. Despite the potential reported in individual studies, methylation markers still require validation before being recommended for clinical practice. This systematic review and meta-analysis aimed to evaluate the performance of DNA methylation-based biomarkers for detecting high-grade intraepithelial lesions (HSIL) in hrHPV-positive women.

**Methods:**

Hence, PubMed, Scopus, and Cochrane databases were searched for studies that assessed methylation in hrHPV-positive women in cervical scrapes. Histologically confirmed HSIL was used as endpoint and QUADAS-2 tool enabled assessment of study quality. A bivariate random-effect model was employed to pool the estimated sensitivity and specificity as well as positive (PPV) and negative (NPV) predictive values.

**Results:**

Twenty-three studies were included in this meta-analysis, from which cohort and referral population-based studies corresponded to nearly 65%. Most of the women analyzed were Dutch, and *CADM1, FAM19A4, MAL,* and *miR124-2* were the most studied genes. Pooled sensitivity and specificity were 0.68 (CI 95% 0.63–0.72) and 0.75 (CI 95% 0.71–0.80) for cervical intraepithelial neoplasia (CIN) 2+ detection, respectively. For CIN3+ detection, pooled sensitivity and specificity were 0.78 (CI 95% 0.74–0.82) and 0.74 (CI 95% 0.69–0.78), respectively. For pooled prevalence, PPV for CIN2+ and CIN3+ detection were 0.514 and 0.392, respectively. Furthermore, NPV for CIN2+ and CIN3+ detection were 0.857 and 0.938, respectively.

**Conclusions:**

This meta-analysis confirmed the great potential of DNA methylation-based biomarkers as triage tool for hrHPV-positive women in cervical cancer screening. Standardization and improved validation are, however, required. Nevertheless, these markers might represent an excellent alternative to cytology and genotyping for colposcopy referral of hrHPV-positive women, allowing for more cost-effective screening programs.

**Supplementary Information:**

The online version contains supplementary material available at 10.1186/s13148-023-01537-2.

## Background

Currently, cervical cancer remains a significant public health concern at global level. Not only does it represent the fourth most incident malignancy in women (with an age-standardized incidence rate of 13.3 per 100,000 female individuals in 2020, worldwide), but also it is the third most deadly cancer (with an age-standardized mortality rate of 7.3 per 100,000 women in 2020 worldwide) [1]. These figures, nonetheless, hide remarkable geographical differences, with cervical cancer-related deaths being more impressive in countries with low human development index [1]. Although this may be partially explained by limited access to high-quality medical care, lack of effective preventive strategies, including screening, constitutes the major cause. Because cervical cancer is a preventable disease, screening strategies, based on cervical cytology and/or high-risk HPV (hrHPV) testing implemented at younger ages (below 30–35 years), detect with noticeable sensitivity and specificity the precancerous lesions amenable for treatment before overtly invasive cancer develops [2, 3]. The vast majority of cervical cancers are hrHPV-related, and the implication of this virus in cervical cancer pathobiology is well known, namely its effect on the transformation of epithelial surfaces like the squamous–columnar junction of the cervix or the lymph epithelium of the base of tongue and tonsils [4]. In recent years, screening strategies have progressively focused on hrHPV testing as first-line screening test [5–7], owing to its higher sensitivity. However, hrHPV infections detected may also correspond to transient infection, and thus, this test is unable to specifically identify women which really need to be referred for a specialized consultation and undergo colposcopy-guided biopsy, a rather invasive procedure. Indeed, an accurate test which might identify clinically relevant hrHPV infections is key to reduce the number of unneeded referrals and interventions (with associated risks and costs) as well as hrHPV test repetitions [8, 9]. DNA methylation, the most studied epigenetic mechanism involved in gene expression regulation, has been successfully explored as a source of noninvasive disease biomarkers [10, 11]. Specifically, in cervical cancer, shifts in promoter methylation levels of several genes (both human and part of the HPV genome) have been associated with HPV status, lesion progression, and patient outcome [10, 11]. Despite a very promising performance demonstrated in individual studies, the fact is that such methylation-based tests have not moved from research to clinical practice, yet. Importantly, reports in the literature are characterized by heterogeneity of study settings, populations, methodological strategies, technicalities, and cutoff values used, among other variables, hampering a comprehensive overview of these tests’ performance and their real clinical usefulness, as well as the added value of comparing with standard methods like cytology and hrHPV genotyping [3].

In this systematic review and meta-analysis, we aimed to evaluate the performance of DNA methylation-based biomarkers for detecting high-grade intraepithelial lesions (HSIL), i.e., cervical intraepithelial neoplasia (CIN)2 + and CIN3+ in hrHPV-positive women and assess their potential as triage biomarkers in these women, to better ascertain their value in the context of cervical cancer screening. Furthermore, we identified the gaps that still preclude their translation into the clinics.

## Results

### Literature overview

**Fig. 1:**
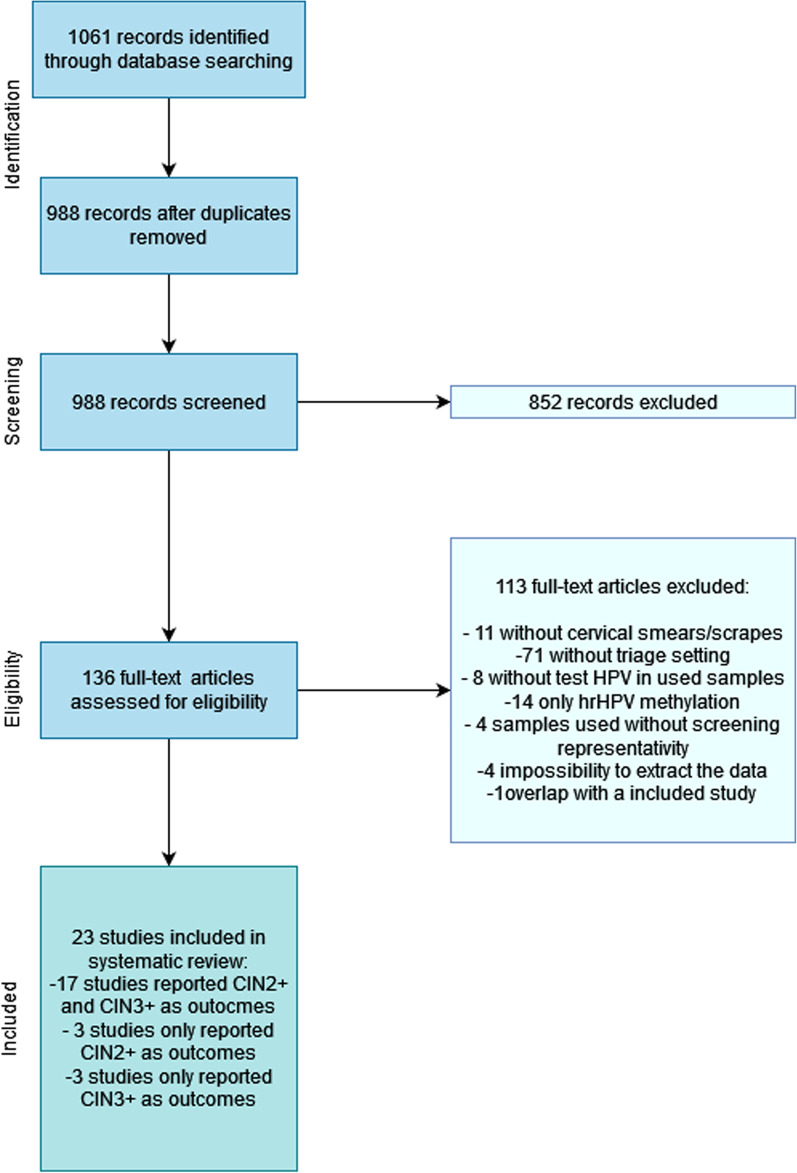
Flowchart of the systematic review and studies included in the meta-analysis

Our search retrieved 536 records in PubMed, 498 in Scopus, and 27 in Cochrane, achieving a total of 1061 records, 73 of which were duplicates (Fig. 1). From the remaining 988 publications, 852 were excluded after abstract and title review. Another 113 publications were further excluded: 11 did not use cervical smears/scrapes; 71 did not perform methylation analysis in a hrHPV-positive women setting (i.e., as triage); 8 studies did not test hrHPV in the samples used; 14 only presented hrHPV methylation; 4 performed DNA methylation analysis in only a subset of samples (e.g., CIN1 vs. CIN3); 4 did not allow for data extraction; and 1 disclosed a high level of overlap with another included study (B1) [12]. Hence, 23 articles were included in the final analysis, and these are summarized in Table 1 and Additional file 1: Table S1.

**Table 1:** Individual studies characterization

ID	Author	Year of study publication	Type of study	Study population origin (country)	Cohort origin	Sample type	Age (range)	Number of samples	Number of CIN2+ (%)	Number of CIN3+ (%)	DNA methylation markers studied	Panel versus single versus both	Platform	Pre-established cutoff yes/no/unclear	Refs.
*Studies with health professional-collected samples*															
L1	Bonde ^a^	2021	Convenience^1^	Scotland	PAVDAG	Cervical scrape	30–61	161	40 (24.8%)	25 (15.5%)	FAM19A4/miR124-2	Panel	QMSP	Yes	[13]
	Bonde ^b^			Denmark	VALGENT4 + routine screening		30–65	424	75 (17.7%)	57 (13.4%)					
	Bonde ^c^			Slovenia	Routine screening		30–76.3	928	102 (11.0%)	60 (6.5%)					
	Bonde ^d^			The Netherlands	VUSA screen		29–61	871	155 (17.8%)	106 (12.2%)					
B1	Hesselink	2011	Convenience^1^	The Netherlands	Routine screening	Cervical scrape	19–62	236	58 (24.6%)	38 (16.1%)	CADM1/MAL	Panel	QMSP	Yes	[12]
F5	Verhoef	2015	Referral population based	The Netherlands	PROHTECT-3 (Cytology arm)	Cervical scrape	38–48	364	90 (24.7%)	62 (17.0%)	CADM1/MAL	Panel	QMSP	Yes	[20]
L2	Vink	2021	Referral population based	The Netherlands	POBASCAM	Cervical scrape	26–61	979	–	115 (11.7%)	FAM19A4/miR124-2	Panel	QMSP	Yes	[23]
F1	Vuyst	2015	Cohort	Kenya	HIV-infected	Cervical scrape	18–55	248	93 (37.5%)	–	CADM1/MAL/miR124-2	Panel	QMSP	No	[33]
I1	Bu	2018	Cohort	China	Routine screening	Cervical scrape	–	215	118 (54.9%)	–	FAM19A4	Single	QMSP	No	[28]
E4	De Strooper	2014	Cohort	The Netherlands	Routine screening	Cervical scrape	19–62	218	52 (23.9%)	33 (15.1%)	FAM19A4	Single	QMSP	Yes	[17]
G4	Luttmer	2016	Referral population based	The Netherlands	COMETH	Cervical scrape	18–70	508	180 (35.4%)	90 (17.7%)	FAM19A4	Single	QMSP	Yes	[18]
J1	Cook	2019	Case–control	Canada	FOCAL	Cervical scrape	25–65	257	107 (41.6%)	44 (17.1%)	S5 (EPB41L3/HPV16L1/HPV16L2/HPV18L2/HPV31L1/HPV33L2)	Panel	QMSP	Yes	[34]
G2	Lorincz	2016	Case–control	United Kingdom	Predictors 3 (P3)	Cervical scrape	–	341	39 (11.4%)	–	S5 (EPB41L3/HPV16L1/HPV16L2/HPV18L2/HPV31L1/HPV33L2) and S4 (EPB41L3/HPV16L1/HPV16L2/HPV18L2/HPV31L1)	Panel	Pyrosequencing	Yes	[25]
E1	Boers	2014	Convenience^1^	The Netherlands	PROHTECT-3B	Cervical scrape	33–67	128	49 (38.3%)	34 (26.6%)	C13ORF18/EPB41L3/JAM3/TERT	Single	QMSP	No	[16]
K3	Li	2020	Cohorts	Chinese	Outpatient clinic population	Cervical scrape	18–67	227	129 (56.8%)	83 (36.6%)	ANKRD18CP/C13orf18/EPB41L3/JAM3/SOX1/ZCAN1	Both	QMSP	Yes	[29]
J7	van Leeuwen	2019	Referral population based	Slovenian	Slovenian HPV Prevalence Study	Cervical scrape	–	235	35 (14.9%)	19 (8.1%)	ANKRD18CP/ C13orf18/EPB41L3/ JAM3/SOX1/ZCAN1	Both	QMSP	Yes	[27]
H7	Yuan	2017	Case–control	China	Hospital-based study	Cervical scrape	–	259	164 (63.3%)	118 (45.6%)	C13ORF18/JAM3/ SLIT2/SOX1/TERT	Both	Pyrosequencing	Unclear	[32]
F6	Yin	2015	Cohort	China	Outpatient clinic population	Cervical scrape	–	168	72 (42.9%)	31 (18.5%)	JAM3	Single	QMSP	No	[31]
E5	Hansel	2014	Cohort	Germany	Outpatient clinic population	Cervical scrape	18–81	217	84 (38.7%)	42 (19.4%)	DLX1/ITGA4/RXFP3/SOX17/ZNF671	Panel	QMSP	No	[24]
H4	Schmitz	2017	Cohort	Germany	Outpatient clinic population	Cervical scrape	–	189	–-	89 (47.1%)	ASTN1/DLX1/ITGA4/RXFP3/SOX17/ ZNF671	Panel	QMSP	Yes	[26]
H5	Tian	2017	Cohort	China	Outpatient clinic population	Cervical scrape	–	312	–-	155 (49.7%)	ZNF582/PAX1	Both^2^	QMSP	Yes	[30]
*Studies with self-collected samples*															
G1	De Strooper ^a^	2016	Referral population based	The Netherlands	PROHTECT-3A	Self-collected cervico-vaginal lavage	33–63	389	119 (30.6%)	78 (20.1%)	FAM19A4/miR124-2	Panel	QMSP	Yes	[14]
	De Strooper ^b^	2016		The Netherlands	PROHTECT-2	Self-collected vaginal brush	30–62	254	99 (39.0%)	72 (28.3%)					
E10	Verhoef	2014	Referral population based	The Netherlands	PROHTECT-3 (methylation arm)	Self-collected cervico-vaginal lavage	30–60	408	124 (30.4%)	79 (19.4%)	MAL/miR124-2	Panel	QMSP	Yes	[21]
E9	Verhoef	2014	Referral population based	The Netherlands	PROHTECT-3	Self-collected cervico-vaginal lavage	30–64	1019	225 (22.1%)	147 (14.4%)	MAL/miR124-2	Panel	QMSP	Yes	[22]
G3	Luttmer	2016	Referral population based	The Netherlands	COMETH	Self-collected cervico-vaginal lavage	18–66	450	153 (34.0%)	75 (16.7%)	FAM19A4	Single	QMSP	Yes	[19]
I8	Verlaat^a^	2018	Convenience^1^	The Netherlands	PROHTECT-3B	Self-collected vaginal brush	27–75	287	109 (38.0%)	81 (28.2%)	ASCL1/LHX8/ ST6GALNAC5	Panel	QMSP	Yes	[15]
	Verlaat^b^	2018		The Netherlands	PROHTECT-3	Self-collected cervico-vaginal lavage	33–63	199	65 (32.7%)	43 (21.6%)					

^1^The study used a selection of samples from a previous population-based study; ^2^ZNF582 was evaluated individually and as panel with PAX1

Among these studies, one analyzed a population from 4 different countries and was thus considered as representing four independent studies [13], and two studies reported two sets of different samples, which were also considered independent [14, 15]. Although most of the studies (12/23) were conducted in the Dutch population [12–23], one also analyzed populations from Scotland, Denmark, and Slovenia [13]. Four additional studies were conducted in European women [24–27], while 5 studies were conducted in the Chinese population [28–32], one in the Canadian population [34], and another in the Kenyan population [33].

Eight articles evaluated DNA methylation of *cell adhesion molecule 1* (*CADM1*), *TAFA chemokine-like family member 4* (*TAFA4,* also known as *FAM19A4*), *mal, T cell differentiation protein* (*MAL*), and *microRNA 124-2* (*miR124-2*) genes in different combinations [12–14, 20–23, 33], whereas four studies only evaluated *FAM19A4* methylation [17–19, 28]. Two studies assessed the performance of the S5 classifier [a methylation panel comprising *erythrocyte membrane protein band 4.1 like 3* (*EPB41L3*), *HPV16L1, HPV16L2, HPV18L2, HPV31L1, HPV33L2*], one of which also included the evaluation of S4 classifier (*EPB41L3*, *HPV16L1, HPV16L2, HPV18L2, HPV31L1*) [25, 34]. *EPBL41L3* methylation levels were assessed in three more studies alone, and different combinations with *ankyrin repeat domain 18C, pseudogene* (*ANKRD18CP*), *rubicon-like autophagy enhancer* (*RUBCNL* also known as *C13orf18*), *junctional adhesion molecule 3* (*JAM3), SRY-box transcription factor 1* (*SOX1*), *telomerase reverse transcriptase* (*TERT*), *zinc finger and SCAN domain containing* 1 (*ZCAN1*) [16, 27, 29]. *C13orf18, JAM3, SOX1,* and *TERT* were evaluated individually and in panels with different combinations of two genes, including *slit guidance ligand 2* (*SLIT2*) gene [32]. One study evaluated *JAM3* methylation individually [31]. Two more studies focused on methylation of *distal-less homeobox 1* (*DLX1*), *integrin subunit alpha 4* (ITGA4), *relaxin family peptide receptor 3* (*RXFP3*), *SRY-box transcription factor 17* (*SOX17*), and *zinc finger protein 671* (*ZNF671*), and other also evaluated the methylation of *ANKRD18CP*. Both studies assessed the genes individually and as a panel [24, 26]. Furthermore, one study reported methylation levels of *zinc finger protein 582* (*ZNF582*) individually and as part of a panel with *paired box 1* (*PAX1*) [30]. Lastly, one study assessed the methylation levels of *achaete-scute family bHLH transcription factor 1* (*ASCL1*), *LIM homeobox 8* (*LHX8*), and *ST6 N-acetylgalactosaminide alpha-2,6-sialyltransferase 5* (*ST6GALNAC5*) in a panel [15].

Overall, 17 (74%) studies reported DNA methylation markers performance for both outcomes considered in this systematic review and meta-analysis (CIN2+ and CIN3+) [12–22, 24, 27, 29, 31, 32, 34]. Three (13%) studies only reported DNA methylation-based markers performance for CIN2+ outcome [25, 28, 33], and three (13%) more only reported DNA methylation-based markers performance for CIN3+ outcome [23, 26, 30]. Of note, 5 (22%) studies were conducted in self-collected samples [14, 15, 19, 21, 22]. Moreover, eight studies (35%) were referral population-based studies [14, 18–23, 27], eight studies (35%) were cohort studies [17, 24, 26, 28–31, 33], three (13%) were case–control studies [25, 32, 34], and four (17%) were convenience studies [12, 13, 15, 16].

Concerning methylation cutoffs, seventy-three percent of the studies disclosed a predefined cutoff for positivity [12–15, 17–23, 25–30, 34], mostly established through receiver operating characteristics (ROC) curve analysis in a training set or previous studies.

### Quality assessment

**Fig. 2:**
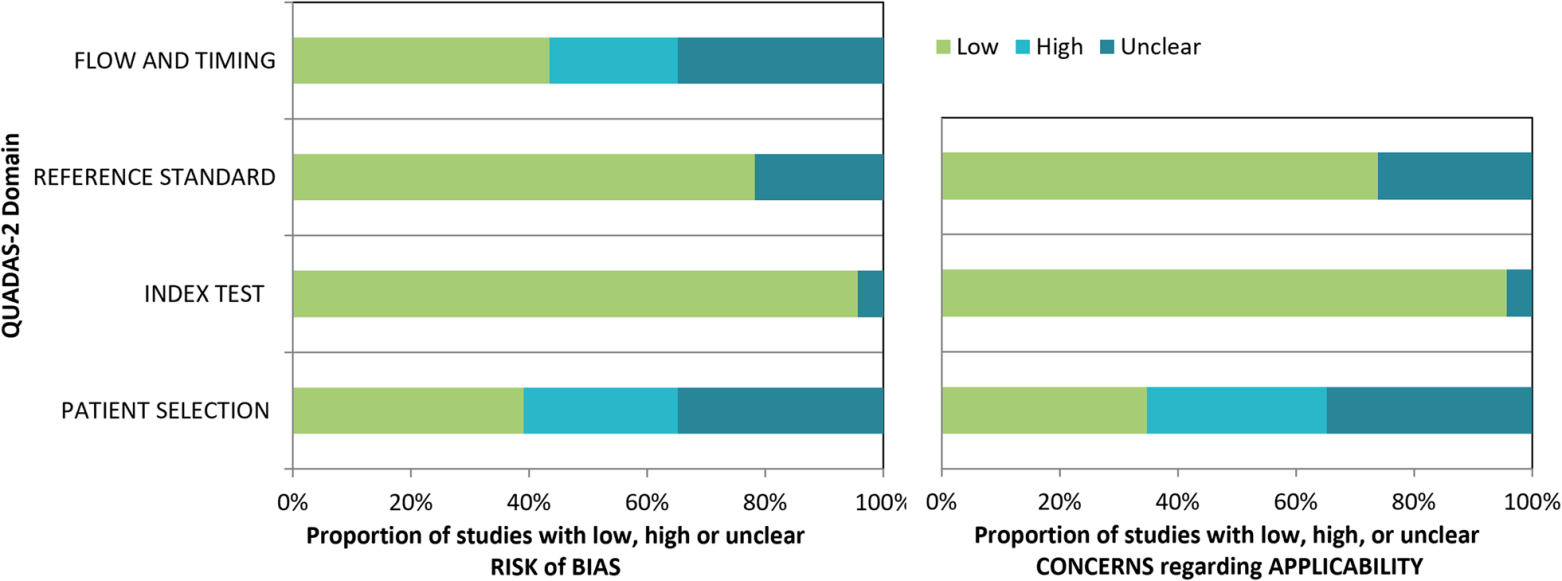
Quality assessment of the individual studies using QUADAS-2 tool. The left panel depicts the risk of bias of the studies and the right panel the risk of concerns regarding applicability

The quality of individual studies was assessed using QUADAS-2 and is summarized in Fig. 2 and Additional file 1: Table S2. The primary source of bias was patient selection. About 20% showed high patient selection bias, which was mainly associated with the design of the primary study. Nonetheless, the triage setting reported by some studies as a secondary outcome might also have led to bias.

Moreover, studies with high patient selection bias were associated with an enrichment of CIN2+ and or CIN3+ lesions, thus not representing a real population-based scenario. Most studies did not fully describe the women lost for follow-up or the interval between the methylation test and the reference test. Studies with high selection bias were removed from some of the analyses to evaluate the impact on biomarker performance (*n* = 7).

### Diagnostic accuracy in an hrHPV-positive women triage setting

**Fig. 3:**
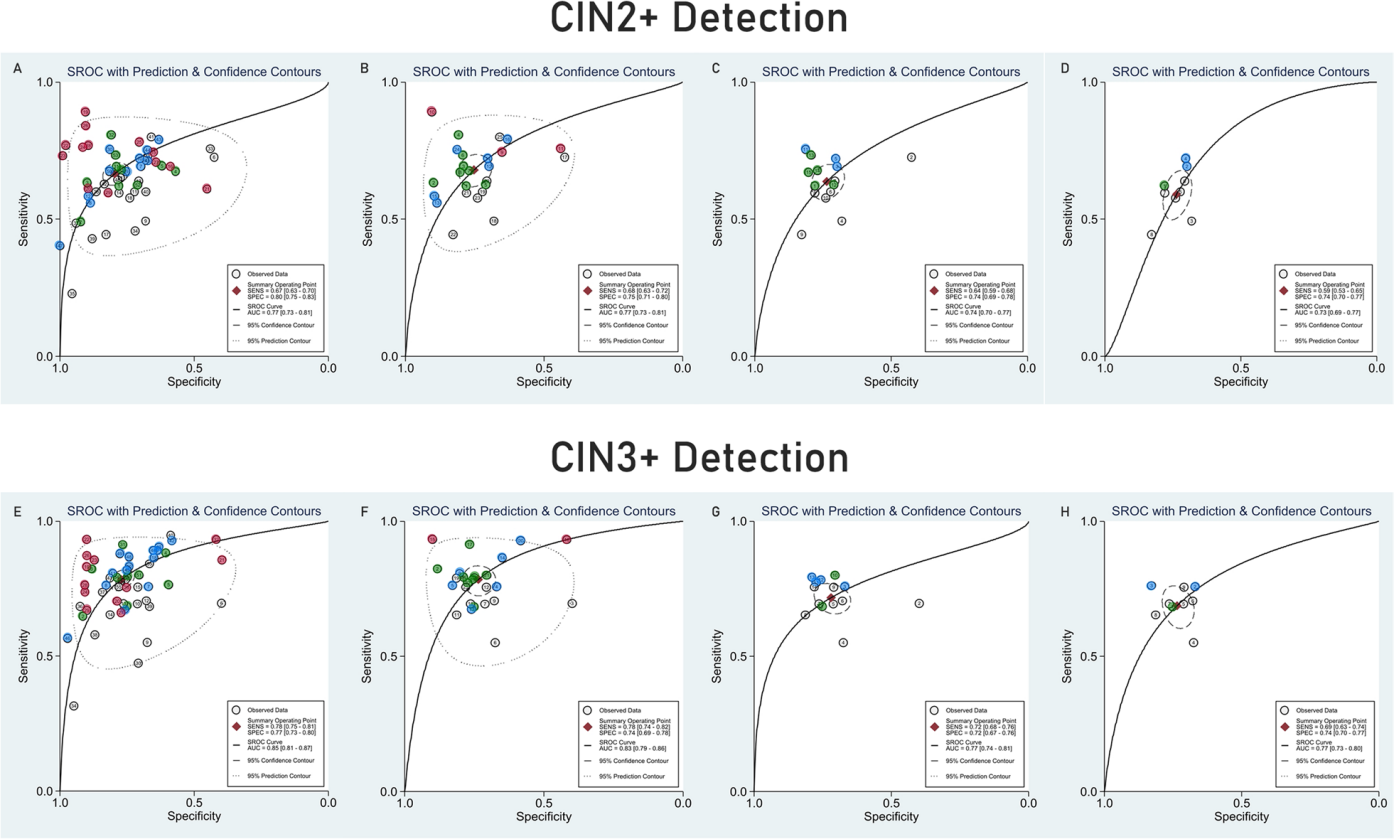
SROC graphs for CIN 2 + (**A**–**D**) and CIN3+ detection (**E**–**H**). **A** and **E** all the markers reported in all studies; **B** and **F** the best markers reported in each study (avoiding considering the same sample more than once); **C**–**G** the most studied genes (*CADM1, FAM19A4, MAL,* and *miR124-2*); and **D**-**H** studies which set the threshold to achieve 70% specificity. Gray dots represent referral population-based studies, blue dots represent cohort studies, red dots represent case–control studies, and green dots represent convenience studies

Diagnostic performance of the reported methylation markers was assessed, and the main performance indicators are depicted in Table 2. For both outcomes, CIN2+ and CIN3+ , sensitivity, specificity, and summary receiver operating characteristic (SROC) curve (Fig. 3) were pooled for: i) all the markers reported in all studies; ii) the best markers reported in each study (avoiding considering the same sample more than once); iii) the most frequently studied genes (*CADM1, FAM19A4, MAL* and *miR124-2*); and iv) studies which set the threshold to achieve 70% specificity. For CIN2+ detection, the pooled AUC was above 73% in all analysis models. Additionally, to evaluate the impact of bias, for analysis ii), high-bias studies were removed, and the outcomes were predicted (Table 2).

**Table 2:** Meta-analysis of the performance of DNA methylation assays for the detection of CIN2+ and CIN3+

	N datasets	N samples	Pooled prevalence	Pooled Sensitivity, (95% CI)	Pooled Specificity, (95% CI)	Q (*p-value*)	I2 (95% CI)	Pooled AUC, (95% CI)
*CIN2* + *detection*								
All studies with all markers ^a,b^[12–22, 24, 25, 27–29, 31–34]	55	16,022	0.35	0.67 (0.63–0.70)	0.80 (0.75–0.83)	422.771 (*p* < 0.001)	100 (99–100)	0.77 (0.73–0.81)
All studies with best markers ^a,c^[12–22, 24, 25, 27–29, 31–34]	25	9011	0.28	0.68 (0.63–0.72)	0.75 (0.71–0.80)	160.418 (*p* < 0.001)	99 (98–99)	0.77 (0.73–0.81)
Studies with *CADM1, FAM19A4, MAL* and *miR124-2*^*a*^[12–14, 17–22, 28, 33]	15	6693	0.25	0.64 (0.59–0.68)	0.74 (0.69–0.78)	77.518 (*p* < 0.001)	97 (96–99)	0.74 (0.70–0.77)
Set threshold to achieve 70% specificity[12, 14, 17–20, 22, 33]	9	3686	0.29	0.59 (0.53–0.65)	0.74 (0.70–0.77)	17.604(*p* < 0.001)	89 (77–100)	0.73 (0.69–0.77)
All studies with best markers excluding high-bias studies ^a,d^[12–15, 17–22, 24, 25, 27, 29]	20	7736	0.25	0.66 (0.61–0.70)	0.74 (0.69–0.78)	110.195 (*p* < 0.001)	98 (97–99)	0.75 (0.71- 0.79)
*CIN3* + *detection*								
All studies with all markers ^a,b^[12–24, 26, 27, 29–32, 34]	55	16,669	0.24	0.78 (0.75–0.81)	0.77 (0.73–0.80)	353.267(*p* < 0.001)	99 (99–100)	0.85 (0.81–0.87)
All studies with best markers ^a,e^[12–24, 26, 27, 29–32, 34]	25	9682	0.18	0.78 (0.74–0.82)	0.74 (0.69–0.78)	87.682 (*p* < 0.001)	98 (96–99)	0.83 (0.79 -0.86)
Studies with *CADM1, FAM19A4, MAL* and *miR124-2*^a^ [12–14, 17–23]	14	7202	0.14	0.72 (0.68–0.76)	0.72 (0.67–0.76)	24.008 (*p* < 0.001)	92 (84–99)	0.77 (0.74–0.81)
Set threshold to achieve 70% specificity [12, 14, 17–20, 22, 33]	9	3655	0.17	0.69 (0.63–0.74)	0.74 (0.70–0.77)	2.778 (*p* = 0.125)	28 (0–100)	0.77 (0.73–0.80)
All studies with best markers excluding high-bias studies ^a,f^[12–15, 17–24, 27, 29, 30]	20	8681	0.17	0.77 (0.72–0.81)	0.73 (0.68–0.76)	58.123 (*p* < 0.001)	97 (94–99)	0.81 (0.77- 0.84)

^a^The sensitivity and specificity were estimated as reported by authors. When multiple thresholds reported, 70% specificity was selected. ^b^Pooled together all the genes and gene combination reported in each study. For studies [16, 23, 25, 26, 29, 32], more than one entrance was considered. ^c^Only one entrance per study was considered. The best combination reported by the authors was selected. For [16] and [32] was considered *JAM3*; for [29] and [27] was considered *C13orf18/EPB41L3/JAM3*; for [25] was considered S5 classifier (*EPB41L3/HPV16L1/HPV16L2/HPV18L2/HPV31L1/HPV33L2*). ^d^Only one entrance per study was considered. The best combination reported by the authors was selected. For [29] and [27] was considered *C13orf18/EPB41L3/JAM3*; for [25] was considered S5 classifier (*EPB41L3/HPV16L1/HPV16L2/HPV18L2/HPV31L1/HPV33L2*). ^e^Only one entrance for study was considered. The best combination reported by the authors was selected. For [30] was considered *PAX1/ZNF582*; for [16] was considered *JAM3*; for [29] was considered *C13orf18/EPB41L3/JAM3*; for [25] was considered S5 classifier; for [27] was considered *SOX1/ZSCAN1*; for [32] was considered *SOX1*. ^f^ Only one entrance for study was considered. The best combination reported by the authors was selected. For [30] was considered *PAX1/ZNF582*; for [29] was considered *C13orf18/EPB41L3/JAM3*; for [25] was considered S5 classifier; for [27] was considered *SOX1/ZSCAN1*; for [32] was considered *SOX1*

Considering only one entrance for the study, sensitivity and specificity for CIN2+ detection were 68% (CI 95% 63–72%) and 75% (CI 95% 71–80%), respectively. Furthermore, for CIN3+ detection, the pooled AUC was higher than 77% in all groups. For the best markers of each study, sensitivity reached 78% (CI 95% 74–82%), and 74% (CI 95% 69–78%) specificity was achieved. The pooled sensitivity and specificity of each study are further represented as a forest plot in Additional file 2: Figs. S1 and S2. When studies were stratified by study type, sensitivity and specificity were similar (Table 3).

**Table 3:** Meta-analysis of the performance of DNA methylation assays for the detection of CIN2+ and CIN3+ according to the study design

	N datasets	N samples	Pooled prevalence	Pooled sensitivity (95% CI)	Pooled specificity (95% CI)	Q	I2	Pooled AUC,
						(*p-value*)	(95% CI)	(95% CI)
*CIN2* + *detection* ^a,b,c^								
Population Referral studies [14, 18–22, 27]	8	3627	0.28	0.60 (0.52–0.66)	0.70 (0.62–0.77)	91.489 (*p* < *0.001)*	98 (96–99)	0.68 (0.64–0.72)
Cohort studies [17, 24, 28, 29, 31, 33]	6	1293	0.42	0.70 (0.62–0.76)	0.78 (0.69–0.86)	35.657 (*p* < *0.001*)	94 (90–99)	0.79 (0.75–0.82)
Convenience studies [12, 13, 15, 16]	8	3234	0.20	0.69 (0.64–0.73)	0.79 (0.77–0.81)	0.478 (*p* = 0.394)	0 (0–100)	0.82 (0.78–0.85)
*CIN3* + *detection* ^a,b,d^								
Population referral studies [14, 18–23, 27]	9	4601	0.16	0.69 (0.64–0.74)	0.71 (0.64–0.78)	28.0.19 (*p* < *0.001*)	93 (86–99)	0.75 (0.71–0.78)
Cohort studies [17, 24, 26, 29–31]	6	1331	0.36	0.81 (0.72–0.87)	0.72 (0.65–0.79)	27.134 (*p* < *0.001*)	93 (86–99)	0.83 (0.80–0.86)
Convenience studies [12, 13, 15, 16]	8	3234	0.14	0.80 (0.75–0.84)	0.77 (0.75–0.79)	0.646 *(p* = 0.362)	0 (0–100)	0.81 (0.78–0.84)

^a^Due to the limited number of case–control studies (*n* = 3), it was not possible to perform statistical analysis. ^b^The sensitivity and specificity were estimated as reported by authors. When multiple thresholds reported, 70% specificity was selected. Only one entrance per study was considered. The best combination reported by the authors was selected. ^c^Only one entrance per study was considered. The best combination reported by the authors was selected. For [16] was considered *JAM3*; for [29] was considered *C13orf18/EPB41L3/JAM3*. ^d^Only one entrance per study was considered. The best combination reported by the authors was selected. For H5[30] was considered PAX1/ZNF582; for K3 [29] was considered C13orf18/EPB41L3/JAM3; for J7 [27] was considered SOX1/ZSCAN1 selection bias

The same was observed after excluding high-bias studies from the analysis (Table 2). Moreover, all analyzed models, except for model iv for CIN3+ detection, were associated with high and significant heterogeneity (Q with *p-value* ≤ 0.051 and *I*^*2*^ above 89%). A univariable meta-regression showed a significant correlation with cohort overlapping among studies, the use of one methylation panel, and the existence of a predefined cutoff (Additional file 2: Fig. S3) for both CIN2+ and CIN3 detection. Concerning CIN3+ detection, the type of sample (self-collected or not) was also found significant. When separately analyzed, studies with self-collected samples disclosed slightly lower performance for CIN 2 + [sensitivity: 63% (CI 95% 54–72%) vs. 70% (CI 95% 65–74%); specificity: 73% (CI 95% 63–81%) vs 76% (CI 95% 71–81%); AUC: 73% (CI 95% 69–77%) vs. 79% (CI 95% 75–82%)] and CIN3+ detection [sensitivity: 72% (CI 95% 63–80%) vs. 81% (CI 95% 76–85%); specificity: 70% (CI 95% 60–78%) vs. 75% (CI 95% 70–79%); AUC: 77% (CI 95% 74–81%) vs. 85% (CI 95% 82–88%)], comparatively to studies in which samples were collected by health professionals (Table 4).

**Table 4:** Meta-analysis of the performance of DNA methylation assays for the detection of CIN2+ and CIN3+ according the sample collection method

	N Datasets	N samples	Pooled prevalence	Pooled Sensitivity, (95% CI)	Pooled Specificity, (95% CI)	Q	I2	Pooled AUC,
						(*p-value*)	(95% CI)	(95% CI)
*CIN2* + *detection* ^a,b^								
Health professional-collected [12, 13, 16–18, 20, 24, 25, 27–29, 31–34]	18	6005	0.27	0.70 (0.65–0.74)	0.76 (0.71–0.81)	74.715 (*p* < *0.001)*	97 (96–99)	0.79 (0.75–0.82)
Self-collected ^(14, 19, 21, 22, 28)^	7	3006	0.30	0.63 (0.54–0.72)	0.73 (0.63–0.81)	84.968 (*p* < *0.001*)	98 (96–99)	0.73 (0.69–0.77)
*CIN3* + *detection* ^a,c^								
Health professional-collected [12, 13, 16–18, 20, 23, 24, 26, 27, 29–32, 34]	18	6676	0.18	0.81 (0.76–0.85)	0.75 (0.70–0.79)	52.628 (*p* < *0.001*)	96 (93–99)	0.85 (0.82–0.88)
Self-collected [14, 15, 19, 21, 22]	7	3006	0.19	0.72 (0.63–0.80)	0.70 (0.60–0.78)	32.355 (*p* < *0.001*)	94 (88–99)	0.77 (0.74–0.81)

^a^The sensitivity and specificity were estimated as reported by authors. When multiple thresholds reported, 70% specificity was selected. ^b^Only one entrance per study was considered. The best combination reported by the authors was selected. For [16] and [32] was considered *JAM3*; for [29] and [27] was considered *C13orf18/EPB41L3/JAM3*; for [25] was considered S5 classifier (*EPB41L3/HPV16L1/HPV16L2/HPV18L2/HPV31L1/HPV33L2*). ^c^Only one entrance for study was considered. The best combination reported by the authors was selected. For [30] was considered *PAX1/ZNF582*; for [16] was considered *JAM3*; for [29] was considered *C13orf18/EPB41L3/JAM3*; for [25] was considered S5 classifier; for [27] was considered *SOX1/ZSCAN1*; for [32] was considered *SOX1*. ^f^ Only one entrance for study was considered. The best combination reported by the authors was selected. For [30] was considered *PAX1/ZNF582*; for [29] was considered *C13orf18/EPB41L3/JAM3*; for [25] was considered S5 classifier; for [27] was considered *SOX1/ZSCAN1*; for [32] was considered *SOX1*

The PPV and NPV values were calculated for all conditions reported above and are displayed in Table 5 and Additional file 2: Fig. S4, according to different prevalence ranges. For CIN2+ detection, the pooled prevalence varied between 25 and 35%, with a PPV between 0.446 and 0.620, and NPV always above 0.80. For a 30% prevalence, PPV ranged from 0.490 to 0.565, with an NPV remaining above 0.80. For CIN3+ detection, the PPV for the pooled prevalence ranged between 0.294 and 0.515, with an NPV above 90% for all conditions. For 20% prevalence, PPV was above 0.87 and NPV above 0.90.

**Table 5:** Pooled positive predictive value and negative predictive value according to prevalence

	N datasets	PPV (mean ± SD)	Set prevalence						
		Pooled prevalence^e^	5% PPV (mean ± SD)	10% PPV (mean ± SD)	20% PPV (mean ± SD)	30% PPV (mean ± SD)	40% PPV (mean ± SD)	50% PPV (mean ± SD)	60% PPV (mean ± SD)
*CIN2* + *detection*									
All studies with all markers ^a,b^ [12–22, 24, 25, 27–29, 31–34]	56	0.620 (0.601–0.639)	0.138 (0.128–0.148)	0.252 (0.237–0.267)	0.432 (0.412–0.452)	0.565 (0.545–0.585)	0.669 (0.651–0.687)	0.752 (0.737–0.767)	0.820 (0.808–0.832)
All studies with best markers^a,c^ [12–22, 24, 25, 27–29, 31–34]	25	0.514 (0.491–0.537)	0.126 (0.116–0.136)	0.233 (0.216–0.250)	0.405 (0.382–0.428)	0.538 (0.515–0.561)	0.644 (0.622–0.666)	0.731 (0.713–0.749)	0.803 (0.788–0.818)
Studies with *CADM1, FAM19A4, MAL* and/or *miR124-2*^*a*^ [12–14, 17–22, 28, 33]	15	0.446 (0.424–0.468)	0.113 (0.104–0.122)	0.212 (0.197–0.227)	0.376 (0.355–0.397)	0.508 (0.486–0.530)	0.616 (0.595–0.637)	0.706 (0.688–0.724)	0.783 (0.768–0.798)
Set threshold to achieve 70% specificity [12, 14, 17–20, 22, 33]	9	0.478 (461–0.494)	0.106 (0.099–0.113)	0.200 (0.189–0.211)	0.359 (0.343–0.375)	0.490 (0.473.0.507)	0.599 (0.582–0.616)	0.691 (0.676–0.706)	0.770 (0.758–0.782)
*CIN3* + *detection*									
All studies with all markers ^a,b^ [12–24, 26, 27, 29–32, 34]	55	0.515 (0.497–0.533)	0.151 (0.142–0.160)	0.273 (0.258–0.288)	0.457 (0.439–0.475)	0.591 (0.573–0.609)	0.692 (0.676–0.708)	0.771 (0758–0.784)	0.835 (0.825–0.845)
All studies with best markers^a,d^ [12–24, 26, 27, 29–32, 34]	25	0.392 (0.371–0.413)	0.134 (0.123–0.145)	0.246 (0.229–0.263)	0.423 (0.401–0.445)	0.557 (0.513–0.579)	0.661 (0.641–0.681)	0.745 (0.734–0.756)	0.814 (0.800–0.828)
Studies with *CADM1, FAM19A4, MAL* and/or *miR124-2*^a^ [12–14, 17–23]	14	0.294 (0.274–0.314)	0.119 (0.109–0.129)	0.221 (0.204–0.238)	0.389 (0.365–0.413)	0.522 (0.497–0.547)	0.629 (0.606–0.652)	0.718 (0.698–0.738)	0.792 0.776–0.808)
Set threshold to achieve 70% specificity [12, 14, 17–20, 22, 33]	9	0.347 (0.326–0.368)	0.120 (0.110–0.130)	0.224 (0.208–0.240)	0.393 (0.371–0.415)	0.526 (0.503–0.549)	0.633 (0.612–0.654)	0.721 (0.703–0.739)	0.795 (0.780–0.810)

	N studies	NPV (mean ± SD)	Set prevalence						
		Pooled prevalence^e^	5% NPV (mean ± SD)	10% NPV (mean ± SD)	20% NPV (mean ± SD)	30% NPV (mean ± SD)	40% NPV (mean ± SD)	50% NPV (mean ± SD)	60% NPV (mean ± SD)
*CIN2* + *detection*									
All studies with all markers ^a,b^[12–22, 24, 25, 27–29, 31–34]	56	0.880 (0.875–0885)	0.978 (0.977–079)	0.954 (0.952–0.956)	0.902 (0.898–0.906)	0.844 (838–850)	0.776 (0.768–0.784)	0.698 (0.688–0.708)	0.607 (0.596–0.618)
All studies with best markers^a,c^[12–22, 24, 25, 27–29, 31–34]	25	0.857 (0.848–0.866)	0.978 (0.976–0.980)	0.954 (0.951–0.957)	0.903 (0.897–0.909)	0.844 (0.834–0.854)	0.777 (0.764–790)	0.699 (0.684–0.714)	0.608 (0.591–0.625)
Studies with *CADM1, FAM19A4, MAL* and *miR124-2*^*a*^[12–14, 17–22, 28, 33]	15	0.858 (0.859–0.866)	0.975 (0.973–0.977)	0.948 (0.945–0.951)	0.890 (0.883–0.897)	0.825 (0.815–0.835)	0.752 (0.739–0.765)	0.669 (0.654–0.684)	0.574 (0.557–0.591)
Set threshold to achieve 70% specificity[12, 14, 17–20, 22, 33]	9	0.815 (0.804–0.826)	0.972 (0.970–0.974)	0.942 (0.938–0.946)	0.878 (0.871–0.885)	0.808 (0.797–0.819)	0.730 (0.716–0.744)	0.643 (0.627–0.659)	0.546 (0.529–0.563)
*CIN3* + *detection*									
All studies with all markers^a,b^[12–24, 26, 27, 29–32, 34]	55	0.916 (0.911–0.921)	0.985 (0.984–0.986)	0.969 (0.967–0.971)	0.933 (0.929–0.937)	0.890 (0.884–0.896)	0.839 (0.830–0.847)	0.776 (0.765–0.787)	0.698 (0.684–0.712)
All studies with best markers^a,d^[12–24, 26, 27, 29–32, 34]	25	0.938 (0.932–0.006	0.984 (0.982–0.986)	0.967 (0.964–0.970)	0.929 (0.922–0.936)	0.885 (0.875–0-895)	0.832 (0.818–8.46)	0.768 (0.750–0.783)	0.688 (0.666–0.710)
Studies with *CADM1, FAM19A4, MAL* and *miR124-2*^a^[12–14, 17–23]	14	0.939 (0.934–0.944)	0.979 (0.977–0.981)	0.958 (0.954–0.962)	0.910 (0.903–0.917)	0.855 (0.844–0.866)	0.791 (0.777–0.805)	0.716 (0.698–0.734)	0.627 (0.607–0.647)
Set threshold to achieve 70% specificity[12, 14, 17–20, 22, 33]	9	0.919 (0.912–0.926)	0.978 (0.976–0.980)	0.955 (0.951–0.959)	0.903 (0.895–0.911)	0.845 (0.833–0.857)	0.778 (0.762–0.794)	0.701 (0.681–0.721)	0.610 (0.588–0.632)

^a^The sensitivity and specificity were estimated as reported by authors. When multiple thresholds reported, 70% specificity was selected. ^b^Pooled together all the genes and gene combination reported in each study. For studies [16, 23, 25, 26, 29, 32], more than one entrance was considered. ^c^Only one entrance per study was considered. The best combination reported by the authors was selected. For [16] and [32] was considered *JAM3*; for [29] and [27] was considered *C13orf18/EPB41L3/JAM3*; for [25] was considered S5 classifier (*EPB41L3/HPV16L1/HPV16L2/HPV18L2/HPV31L1/HPV33L2*). ^d^Only one entrance for study was considered. The best combination reported by the authors was selected. For [30] was considered *PAX1/ZNF582*; for [16] was considered *JAM3*; for [29] was considered *C13orf18/EPB41L3/JAM3*; for [25] was considered S5 classifier; for [27] was considered *SOX1/ZSCAN1*; for [32] was considered *SOX1*. ^e^Presented in Table 2

## Discussion

Primary prevention of cervical cancer with a vaccine against hrHPV was an important step for hrHPV control and eradication. Nonetheless, secondary prevention still plays a crucial role in the reduction of incidence and mortality of cervical cancer, especially among unvaccinated women [35, 36]. First-line hrHPV testing for cervical cancer screening became the gold standard for many European countries at regional or national levels, as proposed by HPV Action Network from European Cancer Organisation in *Viral Protection: Achieving the Possible. A Four-Step Plan for Eliminating HPV Cancers in Europe* [7]. However, this shift challenges the sustainability of screening programs, as the number of women referred to colposcopy significantly increased, mostly due to the limited specificity of hrHPV testing, with the identification of transient infection, and the failure to discriminate lesions with risk of progression from those in regression [37, 38].

In recent years, DNA methylation-based biomarkers have been investigated as potential tools for triage of hrHPV-positive cases, in an attempt to reduce the number of cases referred to colposcopy, avoiding overdiagnosis and consequent overtreatment. However, the evidence supporting the use of triage tests remains limited, as acknowledged by the latest Word Health Organization recommendations [39]. Therefore, we conducted a systematic review with a meta-analysis to better understand the actual value of these DNA methylation-based biomarkers.

One of the major challenges (and limitations) of this meta-analysis results from the fact that distinct markers alone or combined in several panel have been reported along with different methodologies (including genes studied and methodological approach), with only a very small number of studies having used exactly the same protocols. Additionally, in some studies a histological biopsy was not performed when the co-test was negative, which might be a source of bias, although the reported risk of misclassification is rather low [40, 41]. Furthermore, some studies considered the women lost to follow-up as negative for CIN2+ lesion, which might be associated with lesion misclassification and, therefore, might have impacted in the estimated sensitivity and specificity.

Notwithstanding, focusing on the best markers reported from each study, DNA methylation markers reached, overall, a specificity similar to that reported for cytology [using atypical squamous cells of undetermined significance (ASC-US) as cutoff] for CIN2+ detection, although with slightly lower sensitivity [42]. Concerning CIN3+ detection, methylation markers provided higher specificity with equivalent sensitivity, compared to cytology [42]. Indeed, it is widely recognized that upfront knowledge of hrHPV positivity impacts on cytological observation and reporting, usually increasing sensitivity but with a decrease in specificity [42, 43]. Except for visual inspection using acetic acid (VIA), meta-analyses of the other recommended triage strategies (genotyping and cytology) displayed lower or similar specificity than that reported for methylation markers in this meta-analysis. VIA, however, disclosed lower sensitivity for CIN3+ detection [42].

*CADM1, FAM19A4, MAL,* and *miR124-2* are the most commonly reported genes analyzed, although in different combinations. Six of the seven studies designed as referral population-based were conducted with these genes. However, most studies were conducted in the Dutch population, which may limit a broader application in clinical practice. Although *FAM19A4* and *miR124-2* methylation test has already received the *Conformité Européene *In Vitro* Diagnostic* (CE-IVD) label through QIAsure Methylation Test [44], concerns about its sensitivity have hindered its diffusion, especially for CIN2+ detection [13]. Additionally, although GynTect® (comprising *ASTN1*, *DLX1, ITGA4*, *RXFP3, SOX17*, and *ZNF671*) has been approved for clinical practice, it was not yet included in WHO guidelines [39].

Interestingly, Kremer and co-workers reported the association between clinical regression of high-grade CIN and a negative result in the QIAsure Methylation test [44]. In this study, women referred for colposcopy with biopsy-confirmed CIN2/CIN3 were monitored every six months during 24–30 months to evaluate clinical regression or progression of HSIL. Importantly, about 75% of women enrolled were under 35 years, i.e., at reproductive age, in which fertility preservation is of utmost importance [44]. Clinical regression was observed in 58% of recruited women, whereas clinical progression occurred in only 22%. Remarkably, a negative methylation result at baseline was associated with an increased likelihood of clinical regression. When combined with the cytological findings [ASC-US or low-grade squamous intraepithelial lesion (LSIL) or negative HPV16 genotyping (HPV16¯)] clinical regression incidence exceeded 85% [44]. Moreover, a report on risk stratification for hrHPV^+^ women with ASC-US/LSIL from the same team demonstrated that a double positive test (methylation and HPV 16/18 genotyping) associated with higher risk of CIN3^+^ incidence compared to a single positive result (methylation or HPV 16/18 genotyping). Furthermore, a double negative result associated with CIN3^+^ incidence risk under 10% [45]. Thus, methylation analyses might identify cases more likely to endure regression. Importantly, studies with self-collected samples demonstrated a slightly lower performance. Of note, most of these studies were population-based, which might be less prone to bias design. *FAM19A4* methylation showed similar performance in self-collected and heath professional-collected samples [18, 19]. Moreover, the study of Kremer et al. also demonstrated similar test performance for clinician- and self-collected samples, encouraging the adoption of this strategy for recruitment of women non-adherent to screening programs, increasing screening uptake as proposed by HPV Action Network 2 and allowing for fully automated molecular testing pipeline.

Furthermore, Kelly et al. [46] also demonstrated the value of methylation markers for cancer detection (not restricted to hrHPV-positive women). Overall, methylation markers disclosed 63% and 71% sensitivity and 76% and 75% specificity, for CIN2+ and CIN3+ , respectively [46]. These results emphasize the benefit of using methylation markers for cervical cancer screening.

## Conclusions

In conclusion, this systematic review and meta-analysis confirmed that DNA methylation-based markers constitute a promising tool for hrHPV-positive women in cervical cancer screening programs as its higher specificity complements the high sensitivity of hrHPV testing. In addition to decreased overdiagnosis and consequent overtreatment, increasing quality of life and reducing healthcare costs, this strategy may also contribute to decrease pressure upon colposcopy units, improving sustainability and waiting times. Cervical cancer screening program evolution over the decades has been constant, in search of the optimal balance between effectiveness and reliability. Methylation markers may well be the next advancement, improving adhesion, cost-effectiveness, and quality of life.

## Methods

### Study outcomes

Studies which reported DNA methylation according to cervical lesions or sensitivity and specificity of the DNA methylation-based assays for detecting the outcome in the hrHPV-positive women population were included in this meta-analysis. Additionally, a histological endpoint of HSIL or higher [CIN2+ or CIN3+ , which can include carcinoma in situ and invasive cervical carcinoma (ICC)] was required for the study inclusion.

### Search strategy and selection process

PubMed, Scopus, and Cochrane databases were searched for publications until March 31, 2021. No other databases or gray literature was used. The detailed search strategies for the three databases are provided as supplementary material (Additional file 2). All titles and abstracts were screened by two independent authors (SS and JL). Full-text copies of the remaining publications were obtained, and eligibility was assessed by the same two authors. A third author (BM) solved the discrepancies in publication eligibility. Each study was identified with an ID code composed by a letter (corresponding to the publication year) and a sequential number to facilitate the identification of the manuscript by the authors. Missing numbers in the identification correspond to excluded articles.

### Inclusion and exclusion criteria

Since we aimed to evaluate the performance of DNA methylation-based assays as a triage test in hrHPV-based primary cervical cancer screening, we only included studies in which cervical swabs/scrapes from hrHPV-positive women were used. Studies that only employed DNA methylation as a primary setting or as triage after an abnormal cytology result were excluded. Studies that only compared a few groups of lesions [e.g., CIN1 vs. ICC] were excluded as they did not mimic the cervical cancer screening program context. Moreover, studies that reported solely the DNA methylation percentage without any estimation for CIN2+ or CIN3+ detection were excluded, as well as those studies reporting DNA methylation only for one type of hrHPV since the results could not be applied to all hrHPV-positive women in cervical cancer screening programs. Only original studies written in Portuguese or English were included.

### Data collection

From the final list, a standardized form was developed for data collection by two independent authors (SS and JL). Any discrepancies were solved by a third author (BM). Detailed information about collected variables is provided as supplementary material. DNA methylation single markers and/or combinations of markers were considered independently when provided. True positives (TP), false positives (FP), true negatives (TN), and false negatives (FN) were extracted for both outcomes (CIN2+ and CIN3+). When not clearly reported, TP, FP, TN, and FN were calculated based on sensitivity and specificity reported in the corresponding manuscripts, following the formulas: sensitivity = TP/total of cases (TP + FN), and specificity = TN/total of controls (TN + FP). When a discrepancy was found between reported and calculated parameters, or when these performance variables could not be calculated, authors were contacted for clarification. Each study was also classified according with the used referral population (if a population-based selection was performed for sample selection, representing a screening program context), cohort (if sample selection was performed based only on hrHPV status), case–control (if samples selection was based on the histological outcome), or convenience (if the studies used a selection of samples from a previous population-based study).

### Statistical analysis

When available, the TP, FP, TN, and FN were extracted using the predefined cutoff of each study. When multiple cutoffs from the ROC curves were available, a predefined 70% specificity was chosen for TP, FP, TN, and FN assessment. Estimated pooled sensitivity and specificity were calculated using a bivariate model in STATA (metandi and midas). In this approach, sensitivity and specificity are pooled as joined variables considering any correlation that might exist between the variables through a random-effect model [47–49]. Moreover, SROC analysis was performed, and the area under the curve (AUC) was estimated. Additionally, subgroup analyses were made for sensitivity and specificity for CIN2+ and CIN3+ outcomes. Univariate meta-regression was performed for the type of sample (self-collected vs. clinician-collected), cohort overlap among studies, methylation panel (multiple vs. single gene analysis), and the existence of predefined cutoff for methylation levels.

Since prevalence highly influences biomarker performance, PPV and NPV were estimated based on pooled sensitivity and specificity. A bivariate random-effect model (predv_r from mada package) was employed using R software instead of pooled likelihood ratios[50–52]. PPV and NPV values were estimated for prevalence ranging between 5 and 60% for CIN2+ and CIN3+ detection [47, 53].

### Quality assessment

Two authors (SS and JL) assessed the quality of studies using the QUADAS-2 [54] tool. Discrepancies were solved by a third author (BM). Bias was assessed based on: participant selection (population characteristics, inclusion and exclusion criteria, and proportion of women with CIN2+ /CIN3+ included), index test description (DNA methylation assessment description and cutoff for methylation positivity), and reference test (histological confirmation assessment). Additional file 1: Table S2 depicts the quality assessment for all the included studies.

This analysis is reported according to the Preferred Reporting Items of Systematic Reviews and Meta-analysis of Diagnostic Test Accuracy Studies (PRISMA-DTA) guidelines [55, 56] and the present review was registered on the PROSPERO database at the Centre of Reviews and Dissemination, University of York, UK, with the registration number CRD42022350086 (https://www.crd.york.ac.uk/PROSPERO/display_record.php?RecordID=350086).

## Supplementary Information

**Additional file 1.** Additional Tables S1–S2.

**Additional file 2.** Additional Figures S1–S4 and Additional Methods.

## Data Availability

All data generated or analyzed during this study are included in this article.

## Abbreviations

ANKRD18CP: Ankyrin repeat domain 18C, pseudogene
ASCL1: Achaete-scute family bHLH transcription factor 1
ASC-US: Atypical squamous cells of undetermined significance
AUC: Area under the curve
CADM1: Cell adhesion molecule 1
CE-IVD: Conformité Européene in vitro diagnostic
CIN: Cervical intraepithelial neoplasia
DLX 1: Distal-less homeobox 1
EPB41L3: Erythrocyte membrane protein band 4.1 like 3
FN: False negatives
FP: False positives
hrHPV: High-risk human papillomavirus
HSIL: High-grade intraepithelial lesions
ICC: Invasive cervical carcinoma
ITGA4: Integrin subunit alpha 4
JAM3: Junctional adhesion molecule 3
LHX8: LIM homeobox 8
MAL: Mal, T cell differentiation protein
miR124-2: MicroRNA 124-2
NPV: Negative predictive values
PAX1: Paired box 1
PPV: Positive predictive values
ROC: Receiver operating characteristics
RUBCNL: Rubicon-like autophagy enhancer
RXFP3: Relaxin family peptide receptor 3
SLIT2: Slit guidance ligand 2
SOX 17: SRY-box transcription factor 17
SOX1: SRY-box transcription factor 1
SROC: Summary receiver operating characteristics
ST6GALNAC5: ST6 N-acetylgalactosaminide alpha-2,6-sialyltransferase 5
TAFA4: TAFA chemokine-life family member 4
TERT: Telomerase reverse transcriptase
TN: True negatives
TP: True positives
ZCAN1: Zinc finger and SCAN domain containing 1
ZNF582: Zinc finger protein 582
ZNF671: Zinc finger protein 671

## References

[1] Sung H, Ferlay J, Siegel RL, Laversanne M, Soerjomataram I, Jemal A (2021). Global cancer statistics 2020: GLOBOCAN estimates of incidence and mortality worldwide for 36 cancers in 185 Countries. CA Cancer J Clin.

[2] Saslow D, Solomon D, Lawson HW, Killackey M, Kulasingam SL, Cain J (2012). American Cancer Society, American Society for Colposcopy and Cervical Pathology, and American Society for Clinical Pathology screening guidelines for the prevention and early detection of cervical cancer. CA Cancer J Clin.

[3] Kyrgiou M, Arbyn M, Bergeron C, Bosch FX, Dillner J, Jit M (2020). Cervical screening: ESGO-EFC position paper of the European Society of Gynaecologic Oncology (ESGO) and the European Federation of Colposcopy (EFC). Br J Cancer.

[4] Cohen PA, Jhingran A, Oaknin A, Denny L (2019). Cervical cancer. Lancet.

[5] Ronco G, Dillner J, Elfstrom KM, Tunesi S, Snijders PJ, Arbyn M (2014). Efficacy of HPV-based screening for prevention of invasive cervical cancer: follow-up of four European randomised controlled trials. Lancet.

[6] Wright TC, Stoler MH, Behrens CM, Sharma A, Zhang G, Wright TL (2015). Primary cervical cancer screening with human papillomavirus: end of study results from the ATHENA study using HPV as the first-line screening test. Gynecol Oncol.

[7] European Cancer Organisation. Viral protection: achieving the possible. A four step plan for eliminating HPV cancers in Europe. 2020.

[8] Kaljouw S, Jansen EEL, Aitken CA, Harrijvan LM, Naber SK, de Kok I (2021). Reducing unnecessary referrals for colposcopy in hrHPV-positive women within the Dutch cervical cancer screening programme: a modelling study. Gynecol Oncol.

[9] Wright TC, Behrens CM, Ranger-Moore J, Rehm S, Sharma A, Stoler MH (2017). Triaging HPV-positive women with p16/Ki-67 dual-stained cytology: results from a sub-study nested into the ATHENA trial. Gynecol Oncol.

[10] Lorincz AT (2016). Virtues and weaknesses of DNA methylation as a test for cervical cancer prevention. Acta Cytol.

[11] Guzel C, van Sten-Van't HJ, de Kok I, Govorukhina NI, Boychenko A, Luider TM (2021). Molecular markers for cervical cancer screening. Expert Rev Proteomics.

[12] Hesselink AT, Heideman DA, Steenbergen RD, Coupe VM, Overmeer RM, Rijkaart D (2011). Combined promoter methylation analysis of CADM1 and MAL: an objective triage tool for high-risk human papillomavirus DNA-positive women. Clin Cancer Res Off J Am Assoc Cancer Res.

[13] Bonde J, Floore A, Ejegod D, Vink FJ, Hesselink A, van de Ven PM (2021). Methylation markers FAM19A4 and miR124-2 as triage strategy for primary human papillomavirus screen positive women: a large European multicenter study. J Int Cancer.

[14] De Strooper LMA, Verhoef VMJ, Berkhof J, Hesselink AT, de Bruin HME, van Kemenade FJ (2016). Validation of the FAM19A4/mir124-2 DNA methylation test for both lavage- and brush-based self-samples to detect cervical (pre)cancer in HPV-positive women. Gynecol Oncol.

[15] Verlaat W, Snoek BC, Heideman DAM, Wilting SM, Snijders PJF, Novianti PW (2018). Identification and validation of a 3-gene methylation classifier for HPV-based cervical screening on self-samples. Clin Cancer Res Off J Am Assoc Cancer Res.

[16] Boers A, Bosgraaf RP, van Leeuwen RW, Schuuring E, Heideman DA, Massuger LF (2014). DNA methylation analysis in self-sampled brush material as a triage test in hrHPV-positive women. Br J Cancer.

[17] De Strooper LM, Meijer CJ, Berkhof J, Hesselink AT, Snijders PJ, Steenbergen RD (2014). Methylation analysis of the FAM19A4 gene in cervical scrapes is highly efficient in detecting cervical carcinomas and advanced CIN2/3 lesions. Cancer Prev Res.

[18] Luttmer R, De Strooper LM, Berkhof J, Snijders PJ, Dijkstra MG, Uijterwaal MH (2016). Comparing the performance of FAM19A4 methylation analysis, cytology and HPV16/18 genotyping for the detection of cervical (pre)cancer in high-risk HPV-positive women of a gynecologic outpatient population (COMETH study). J Int Cancer.

[19] Luttmer R, De Strooper LM, Dijkstra MG, Berkhof J, Snijders PJ, Steenbergen RD (2016). FAM19A4 methylation analysis in self-samples compared with cervical scrapes for detecting cervical (pre)cancer in HPV-positive women. Br J Cancer.

[20] Verhoef VM, van Kemenade FJ, Rozendaal L, Heideman DA, Bosgraaf RP, Hesselink AT (2015). Follow-up of high-risk HPV positive women by combined cytology and bi-marker CADM1/MAL methylation analysis on cervical scrapes. Gynecol Oncol.

[21] Verhoef VM, Bosgraaf RP, van Kemenade FJ, Rozendaal L, Heideman DA, Hesselink AT (2014). Triage by methylation-marker testing versus cytology in women who test HPV-positive on self-collected cervicovaginal specimens (PROHTECT-3): a randomised controlled non-inferiority trial. Lancet Oncol.

[22] Verhoef VM, Heideman DA, van Kemenade FJ, Rozendaal L, Bosgraaf RP, Hesselink AT (2014). Methylation marker analysis and HPV16/18 genotyping in high-risk HPV positive self-sampled specimens to identify women with high grade CIN or cervical cancer. Gynecol Oncol.

[23] Vink FJ, Lissenberg-Witte BI, Meijer C, Berkhof J, van Kemenade FJ, Siebers AG (2021). FAM19A4/miR124-2 methylation analysis as a triage test for HPV-positive women: cross-sectional and longitudinal data from a Dutch screening cohort. Clin Microbiol Infect.

[24] Hansel A, Steinbach D, Greinke C, Schmitz M, Eiselt J, Scheungraber C (2014). A promising DNA methylation signature for the triage of high-risk human papillomavirus DNA-positive women. PLoS One.

[25] Lorincz AT, Brentnall AR, Scibior-Bentkowska D, Reuter C, Banwait R, Cadman L (2016). Validation of a DNA methylation HPV triage classifier in a screening sample. J Int Cancer.

[26] Schmitz M, Wunsch K, Hoyer H, Scheungraber C, Runnebaum IB, Hansel A (2017). Performance of a methylation specific real-time PCR assay as a triage test for HPV-positive women. Clin Epigenet.

[27] van Leeuwen RW, Ostrbenk A, Poljak M, van der Zee AGJ, Schuuring E, Wisman GBA (2019). DNA methylation markers as a triage test for identification of cervical lesions in a high risk human papillomavirus positive screening cohort. J Int Cancer.

[28] Bu Q, Wang S, Ma J, Zhou X, Hu G, Deng H (2018). The clinical significance of FAM19A4 methylation in high-risk HPV-positive cervical samples for the detection of cervical (pre)cancer in Chinese women. BMC Cancer.

[29] Li N, Hu Y, Zhang X, Liu Y, He Y, van der Zee AGJ (2021). DNA methylation markers as triage test for the early identification of cervical lesions in a Chinese population. Int J Cancer.

[30] Tian Y, Yuan Wu NY, Liou YL, Yeh CT, Cao L, Kang YN (2017). Utility of gene methylation analysis, cytological examination, and HPV-16/18 genotyping in triage of high-risk human papilloma virus-positive women. Oncotarget.

[31] Yin A, Zhang Q, Kong X, Jia L, Yang Z, Meng L (2015). JAM3 methylation status as a biomarker for diagnosis of preneoplastic and neoplastic lesions of the cervix. Oncotarget.

[32] Yuan L, Hu Y, Zhou Z, Gong Y, Wang R, Li N (2017). Quantitative methylation analysis to detect cervical (Pre)-cancerous lesions in high-risk HPV-positive women. Int J Clin Exp Med.

[33] De Vuyst H, Franceschi S, Plummer M, Mugo NR, Sakr SR, Meijer CJ (2015). Methylation levels of CADM1, MAL, and MIR124-2 in cervical scrapes for triage of HIV-infected, high-risk HPV-positive women in Kenya. J Acquir Immune Defic Syndr.

[34] Cook DA, Krajden M, Brentnall AR, Gondara L, Chan T, Law JH (2019). Evaluation of a validated methylation triage signature for human papillomavirus positive women in the HPV FOCAL cervical cancer screening trial. Int J Cancer.

[35] Baker P, Kelly D, Medeiros R, Morrissey M, Price R (2021). Eliminating HPV-caused cancers in Europe: achieving the possible. J Cancer Policy.

[36] World Health Organization. Global strategy to accelerate the elimination of cervical cancer as a public health problem. 2020.

[37] Salta S, Maia-Moco L, Estevao-Pereira H, Sequeira JP, Vieira R, Bartosch C (2021). Performance of DNA methylation-based biomarkers in the cervical cancer screening program of northern Portugal: a feasibility study. Int J Cancer.

[38] Loopik DL, Koenjer LM, Siebers AG, Melchers WJG, Bekkers RLM (2021). Benefit and burden in the Dutch cytology-based vs high-risk human papillomavirus-based cervical cancer screening program. Am J Obstet Gynecol.

[39] World Health Organization. WHO guideline for screening and treatment of cervical pre-cancer lesions for cervical cancer prevention. 2021. 34314129

[40] Castle PE, Kinney WK, Xue X, Cheung LC, Gage JC, Zhao FH (2018). Effect of several negative rounds of human papillomavirus and cytology co-testing on safety against cervical cancer: an observational cohort study. Ann Intern Med.

[41] Gage JC, Schiffman M, Katki HA, Castle PE, Fetterman B, Wentzensen N (2014). Reassurance against future risk of precancer and cancer conferred by a negative human papillomavirus test. J Natl Cancer Inst.

[42] World Health Organization. WHO guideline for screening and treatment of cervical pre-cancer lesions for cervical cancer prevention: web annex A: syntheses of evidence. 2021. 34314129

[43] Richardson LA, El-Zein M, Ramanakumar AV, Ratnam S, Sangwa-Lugoma G, Longatto-Filho A (2015). HPV DNA testing with cytology triage in cervical cancer screening: influence of revealing HPV infection status. Cancer Cytopathol.

[44] Kremer WW, Dick S, Heideman DAM, Steenbergen RDM, Bleeker MCG, Verhoeve HR (2022). Clinical regression of high-grade cervical intraepithelial neoplasia is associated with absence of FAM19A4/miR124-2 DNA methylation (CONCERVE Study). J Clin Oncol Off J Am Soc Clin Oncol.

[45] Dick S, Vink FJ, Heideman DAM, Lissenberg-Witte BI, Meijer C, Berkhof J (2022). Risk-stratification of HPV-positive women with low-grade cytology by FAM19A4/miR124-2 methylation and HPV genotyping. Br J Cancer.

[46] Kelly H, Benavente Y, Pavon MA, De Sanjose S, Mayaud P, Lorincz AT (2019). Performance of DNA methylation assays for detection of high-grade cervical intraepithelial neoplasia (CIN2+): a systematic review and meta-analysis. Br J Cancer.

[47] Reitsma JB, Glas AS, Rutjes AW, Scholten RJ, Bossuyt PM, Zwinderman AH (2005). Bivariate analysis of sensitivity and specificity produces informative summary measures in diagnostic reviews. J Clin Epidemiol.

[48] Harbord RM, Whiting P (2009). Metandi: meta-analysis of diagnostic accuracy using hierarchical logistic regression. Stand Genomic Sci.

[49] Dwamena B, Sylvester R, Carlos R. Midas: meta-analysis of diagnostic accuracy studies. Accessed on February 8, 2017. Available at: http://fmwww.bc.edu/repec/bocode/m/midas.pdf View in Article. 2009.

[50] Zwinderman AH, Bossuyt PM (2008). We should not pool diagnostic likelihood ratios in systematic reviews. Stat Med.

[51] Doebler P. Meta-analysis of diagnostic accuracy (Version 0.5.11) [Software] 2022 Available from: cran.r-project.org/web/packages/mada/mada.pdf.

[52] Doebler P, Holling H (2015). Meta-analysis of diagnostic accuracy with mada. R Packag.

[53] Sousa-Pinto B, Tarrio I, Blumenthal KG, Araujo L, Azevedo LF, Delgado L (2021). Accuracy of penicillin allergy diagnostic tests: a systematic review and meta-analysis. J Allergy Clin Immunol.

[54] Whiting PF, Rutjes AW, Westwood ME, Mallett S, Deeks JJ, Reitsma JB (2011). QUADAS-2: a revised tool for the quality assessment of diagnostic accuracy studies. Ann Intern Med.

[55] Salameh JP, Bossuyt PM, McGrath TA, Thombs BD, Hyde CJ, Macaskill P (2020). Preferred reporting items for systematic review and meta-analysis of diagnostic test accuracy studies (PRISMA-DTA): explanation, elaboration, and checklist. BMJ.

[56] McInnes MDF, Moher D, Thombs BD, McGrath TA, Bossuyt PM, the P-DTAG (2018). Preferred reporting items for a systematic review and meta-analysis of diagnostic test accuracy studies: the PRISMA-DTA statement. JAMA.

