# Supplementary material for: DNA methylation as a triage marker for colposcopy referral in HPV-based cervical cancer screening: a systematic review and meta-analysis
Source: Clin Epigenetics. 2023 Aug 2;15:125. doi: 10.1186/s13148-023-01537-2 (PMC10399027; doi:10.1186/s13148-023-01537-2)
Supplement: Supplementary file 2 — Additional file 2. Additional Figures S1–S4 and Additional Methods. [file 13148_2023_1537_MOESM2_ESM.docx]

**Additional file 2**

**Additional Figures Legends**

**Additional Figure S1 – Forest plot of individual studies for sensitivity (left panel) and specificity (right panel) for CIN2+ detection considering the best marker reported in each study.** Top studies (gray): referral population-based studies; Middle upper (blue): cohort studies; Middle bottom (red): case-control studies; Bottom (green): convenience studies.

**Additional Figure S2 – Forest plot of individual studies for sensitivity (left panel) and specificity (right panel) for CIN3+ detection considering the best marker reported in each study.** Top studies (gray): referral population-based studies; Middle upper (blue): cohort studies; Middle bottom (red): case-control studies; Bottom (green): convenience studies.

**Additional Figure S3 – Univariable meta-regression for CIN 2+ (A-D) and CIN3+ detection (E-H).** (A and E) all the markers reported in all studies; (B and F) the best markers reported in each study (avoiding considering the same sample more than once); (C-G) the most studied genes (*CADM1, FAM19A4, MAL,* and *miR124-2*); and (D-H) studies which set the threshold to achieve 70% specificity.

**Additional Figure S4 – Positive predictive value (PPV) in red and Negative predictive value (NPV) in blue for CIN 2+ (A-D) and CIN3+ detection (E-H).** (A and E) all the markers reported in all studies; (B and F) the best markers reported in each study (avoiding considering the same sample more than once); (C-G) the most studied genes (*CADM1, FAM19A4, MAL,* and *miR124-2*); and (D-H) studies which set the threshold to achieve 70% specificity.

**Figure S1**

**
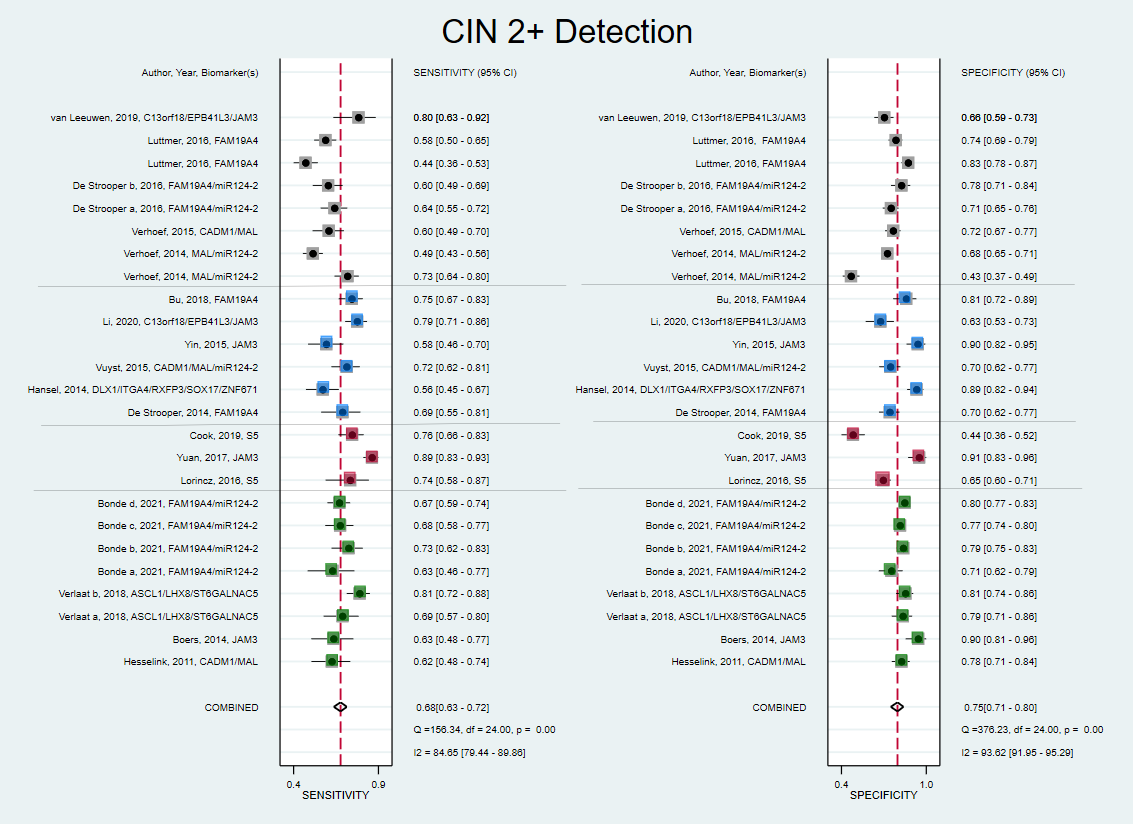
**

**Figure S2**

**
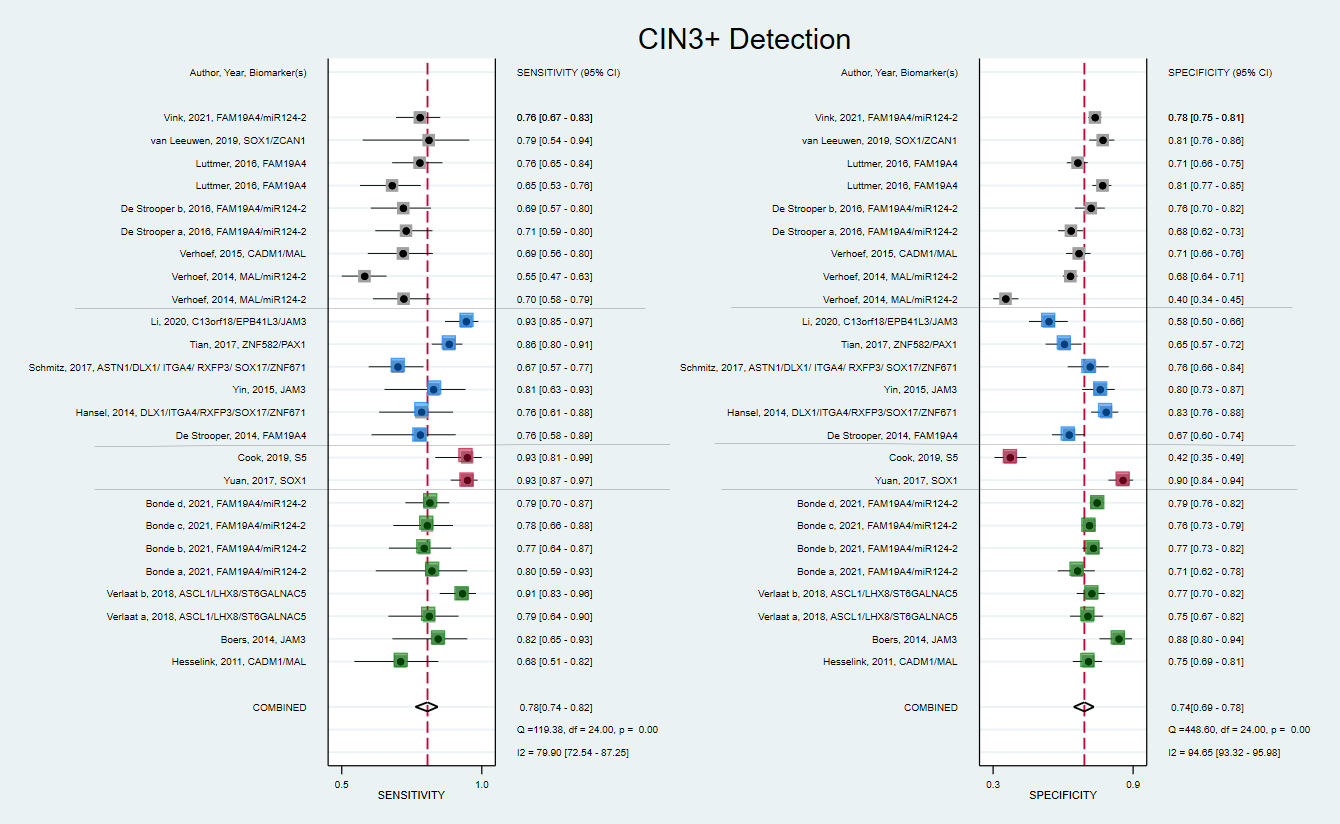
**

**Figure S3**

**
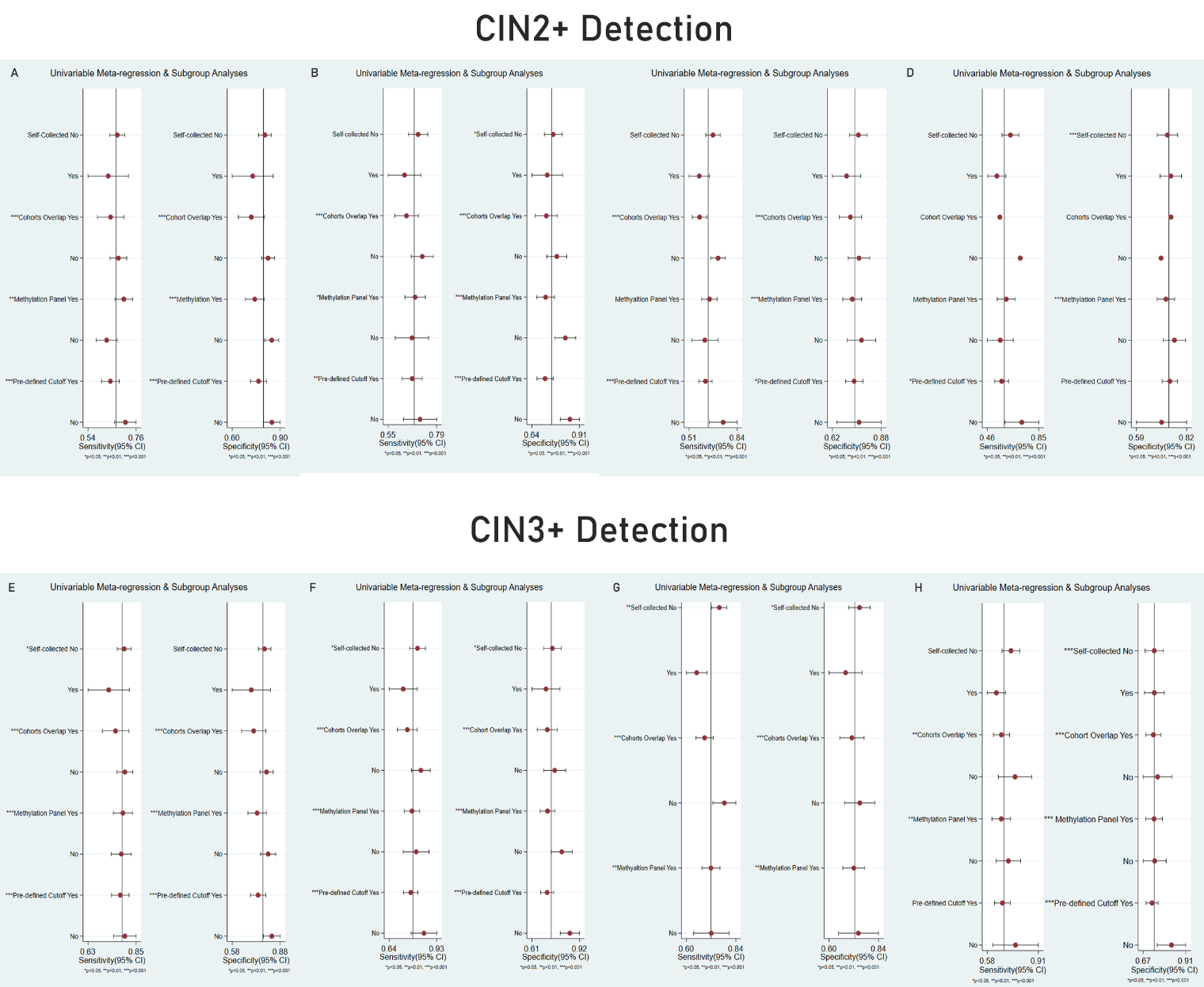
**

**Figure S4**

**
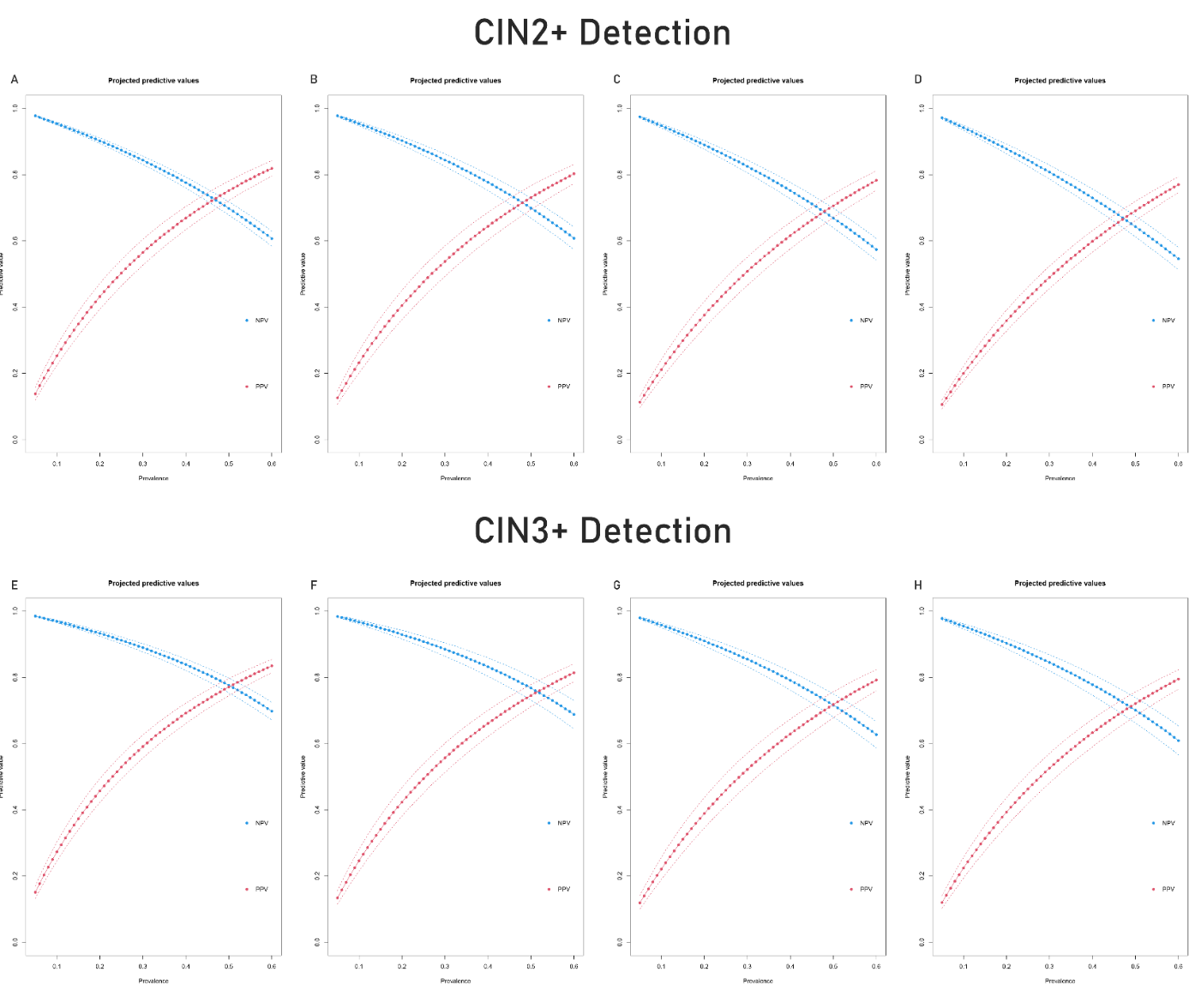
**

**Additional Methods**

**PubMed search strategy**

For PubMed search was used the following strategy: ((("hpv positive" [title/abstract]) OR (“human papillomavirus” [title/abstract]) OR (carcinogenesis [MeSH Terms]) OR ("Cervical Intraepithelial Neoplasia"[Mesh]) OR ("Squamous Intraepithelial Lesions"[Mesh])) AND (("Cervix Uteri"[Mesh]) OR ("Cervix"[title/abstract])) AND ((nucleic acids [MeSH Terms]) OR (CpG islands [MeSH Terms]) OR ("DNA Methylation"[Mesh]) OR ("Epigenomics"[Mesh]) OR (methylation [MeSH Terms])) AND (("Triage"[Mesh]) OR ("Early Detection of Cancer" [Mesh]) OR (“Screening” [title/abstract]) OR ("referral" [title/abstract])))

**Scopus search strategy**

For Scopus search was used the following strategy: ((("hpv positive") OR (“human papillomavirus”) OR (carcinogenesis) OR ("Cervical Intraepithelial Neoplasia") OR ("Squamous Intraepithelial Lesions")) AND (("Cervix Uteri") OR ("Cervix")) AND ((nucleic acids) OR (CpG islands) OR ("DNA Methylation") OR ("Epigenomics") OR (methylation)) AND (("Triage") OR ("Early Detection of Cancer") OR (“Screening”) OR ("referral")))

**Cochrane search strategy**

For Cochrane search was used the following strategy: (((("hpv positive") OR (“human papillomavirus”) OR (carcinogenesis) OR ("Cervical Intraepithelial Neoplasia") OR ("Squamous Intraepithelial Lesions of the cervix")) AND ((nucleic acids) OR (CpG islands) OR ("DNA Methylation") OR ("Epigeneomics") OR (methylation)) AND (("Triage") OR ("Early Detection of Cancer") OR (“Screening”) OR ("referral and consultation")))):ti,ab,kw

**Standardisation of data collection**

Data collected from included studies was performed according to a standardized extraction form. It included: Author, year of publication, country of study population, type of study, number of samples analyzed, age range, cohort name, sample type, number of CIN0/controls, CIN1, CIN2, CIN3,and ICC, DNA methylation marker(s), method of methylation testing, type of cutoff, pre-defined cutoff, population overlap with other studies, number of true positives (TP), true negatives (TN), false positive (FP) and false negatives (FN) for methylation marker(s), cytology and HPV genotyping.
